# Mixed Epithelial Stromal Tumour of the Seminal Vesicle Presenting with Recurrent Acute Urinary Retention: A Case Report

**DOI:** 10.3390/diagnostics16193222

**Published:** 2026-10-05

**Authors:** Daniel Vas, Alessandra Iacono, Tarek Ajami, Silvia Valls, Isabel Trias, Daniel Corominas, Antonio Alcaraz, Carlos Nicolau

**Affiliations:** 1 Department of Radiology, Hospital Clinic de Barcelona, 08036 Barcelona, Spain; dcorominas@clinic.cat (D.C.);; 2 Department of Diagnostic Imaging and Radiation Oncology, Fondazione Policlinico Universitario Agostino Gemelli IRCCS, 00168 Rome, Italy; 3 Department of Urology, Hospital Clínic de Barcelona, 08036 Barcelona, Spain; ajami@clinic.cat (T.A.);; 4 Department of Pathology, Hospital Clínic de Barcelona, 08036 Barcelona, Spain; svallsl@clinic.cat (S.V.);

**Keywords:** mixed epithelial stromal tumour, seminal vesicle, magnetic resonance imaging, retrovesical mass, pelvic mass, urinary retention, cystic neoplasm, radiological-pathological correlation

## Abstract

**Background:** Mixed epithelial stromal tumour (MEST) is a rare biphasic neoplasm, most commonly arising in the kidney. Seminal vesicle involvement is exceedingly uncommon, and its clinical and imaging characteristics remain poorly defined. We present a rare case with detailed clinical and urological evaluation, multiparametric MRI characterisation, and radiologic-pathologic correlation. **Case presentation:** A 34-year-old man presented with recurrent acute urinary retention requiring repeated catheterization and, subsequently, an indwelling urinary catheter. Urological evaluation, including ultrasonography and cystoscopy, demonstrated marked mass effect on the bladder and prostatic urethra without an intravesical lesion. Contrast-enhanced CT and MRI revealed a large retrovesical mass arising from the right seminal vesicle, measuring approximately 11.5 × 9 × 10.5 cm. MRI demonstrated a predominantly multiloculated cystic lesion containing haemorrhagic or proteinaceous material, with an enhancing papillary solid component showing mild diffusion restriction and progressive enhancement. The lesion produced marked mass effect on the bladder, prostate, and rectum, without local invasion. Robotic-assisted resection of the right seminal vesicle was performed. Histopathology demonstrated a biphasic epithelial and stromal proliferation without atypia, necrosis, or increased mitotic activity, consistent with MEST. **Conclusions:** This case highlights the value of integrating clinical, urological, radiological, and pathological findings in the evaluation of a large complex retrovesical mass. Multiparametric MRI provided detailed characterisation of the solid-cystic architecture, with imaging features correlating with the epithelial and stromal components identified histologically. Recognition of this pattern may raise suspicion for MEST and help guide differential diagnosis and surgical planning, although definitive diagnosis requires histopathological and immunohistochemical evaluation.

## 1. Introduction

Mixed epithelial stromal tumour (MEST) is a rare biphasic neoplasm characterised by the coexistence of epithelial structures and stromal components. The most common site of origin for mixed epithelial stromal tumours is the kidney, where they typically present as well-circumscribed mixed solid-cystic renal masses, most often occurring in middle-aged women and frequently characterised by multiloculated cystic components and variable enhancing stromal tissue on imaging [1,2,3]. Extra-renal MESTs have been described in the lower genitourinary tract, including the seminal vesicles, ureter, bladder, prostate, and retroperitoneum, suggesting a shared pathogenetic spectrum of biphasic epithelial–stromal neoplasms [4].

Historically, MESTs have been variably described as epithelial-stromal tumours, or phyllodes-like tumours, reflecting the overlap in histopathological features and evolving nomenclature. As a result, seminal vesicle MESTs are extremely uncommon, with only a limited number of cases available in the literature, and according to the World Health Organization (WHO) Classification, only about a dozen cases of MESTs of the seminal vesicle have been reported with sufficient histopathological description [4,5,6,7,8].

Due to their rarity and non-specific clinical presentation, diagnosis is challenging and often delayed. In this scenario, imaging plays a crucial role in lesion detection and characterisation, as well as in the assessment of local extension and surgical planning. However, the radiological appearance and pathological features may overlap with other retrovesical cystic lesions, including seminal vesicle cystadenoma, Müllerian duct cysts, prostatic cystic neoplasms and malignant tumours. Therefore, the diagnostic process requires careful histological examination, including immunohistochemistry analysis, to rule out other entities with similar imaging characteristics as well as some rare stromo-epithelial prostate lesions [8,9].

## 2. Case Presentation

A 34-year-old man with no relevant past medical history attended multiple urological consultations for recurrent episodes of acute urinary retention requiring repeated bladder catheterisation. Since December 2025, the patient had been carrying an indwelling urinary catheter because of persistent obstructive urinary symptoms and multiple catheter leakages requiring replacement. The patient was referred for further urological evaluation because of a progressively worsening obstructive urinary syndrome associated with a large retrovesical pelvic mass which exerted a significant mass effect on the bladder.

### 2.1. Imaging Findings

**Figure 1:**
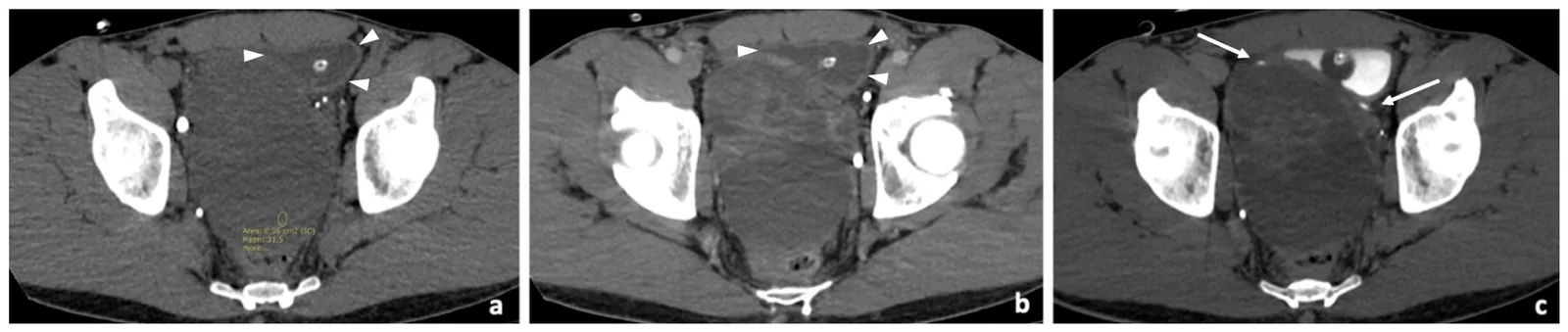
Contrast-enhanced CT demonstrates a predominantly cystic lesion with hyperdense content on the unenhanced acquisition (**a**) and an enhancing solid component on the venous phase (**b**). In all three phases, the urinary bladder is displaced anteriorly (white arrowheads), without evidence of infiltration. On the delayed excretory phase (**c**), the terminal ureters are better delineated (white arrows).

Initial transabdominal suprapubic ultrasound evaluation demonstrated the presence of a space-occupying lesion, apparently arising from the urinary bladder lumen. Serum prostate-specific antigen (PSA) level was normal (0.48 ng/mL). Subsequently, the patient was referred to a contrast-enhanced abdominal CT (Figure 1), which revealed a large heterogeneous mixed solid-cystic pelvic mass measuring approximately 11.5 × 9 × 10.5 cm, located between the bladder and rectum. The lesion appeared predominantly cystic and was initially suspected to arise from the prostate gland. The urinary bladder was displaced anteriorly and showed diffuse wall thickening, likely related to chronic outlet obstruction.

**Figure 2:**
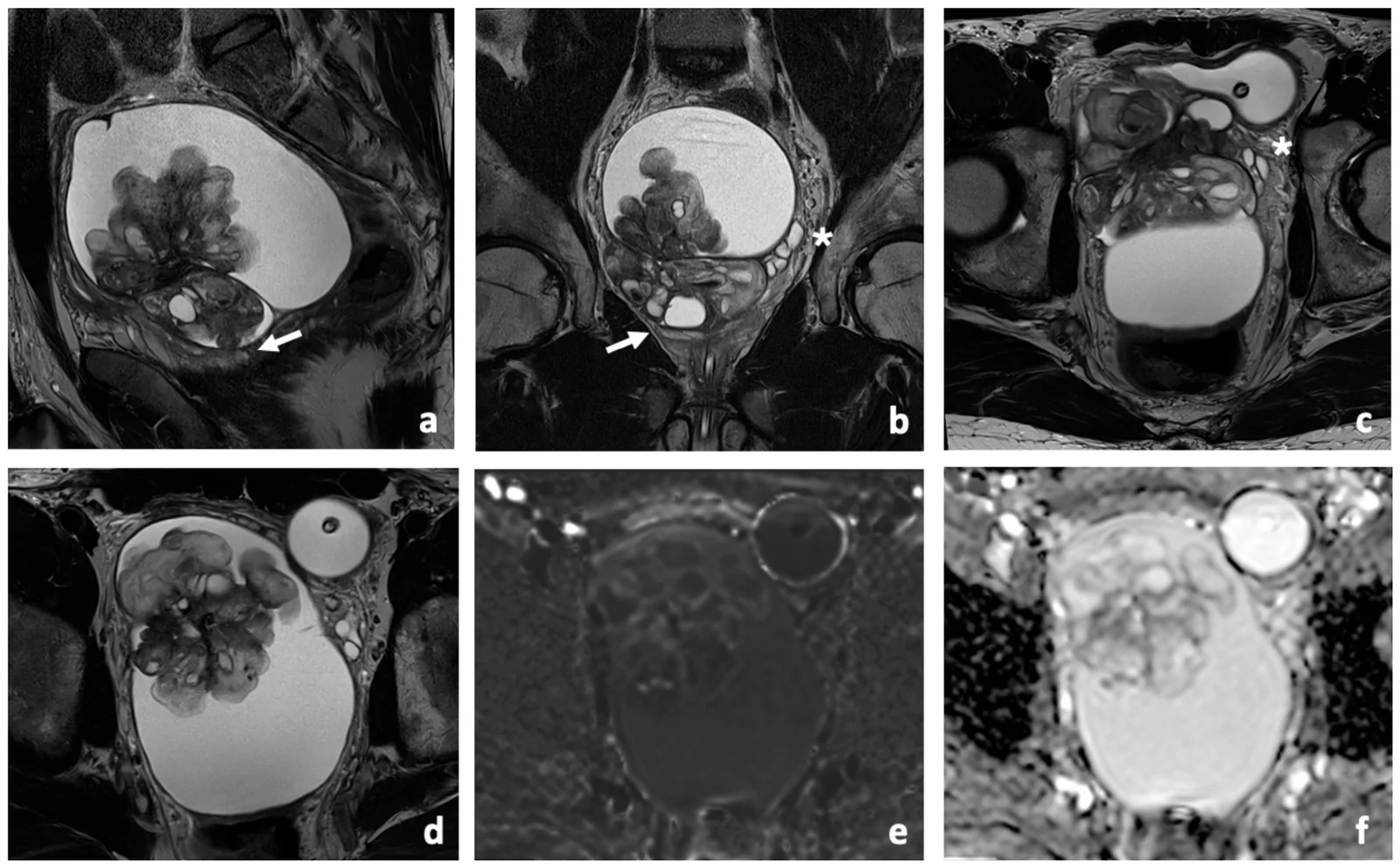
Pelvic MRI showing a large multiloculated retrovesical cystic mass with internal solid papillary component. The lesion exerts marked mass effect on the urinary bladder and the prostate gland (white arrow). Displaced normal left seminal vesicle can be identified (asterisk) on T2WI (**a**–**d**). High b-value DWI (**e**) and ADC map (**f**) demonstrated only slight restricted diffusion of the solid component.

A pelvic MRI was requested to better characterise the lesion. MRI scan demonstrated a large complex retrovesical lesion with mixed solid and cystic components (Figure 2).

**Figure 3:**
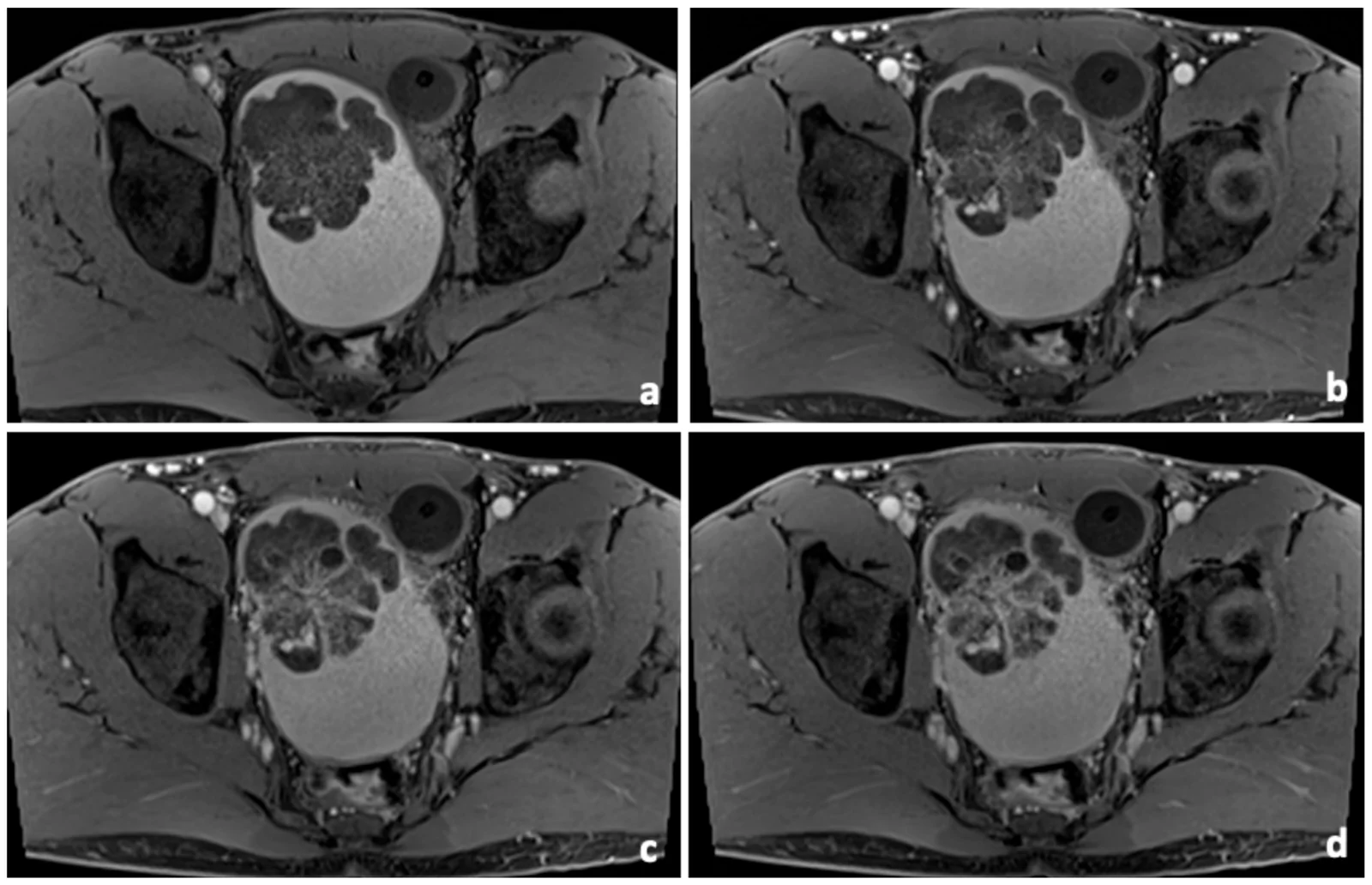
Fat-suppressed T1 image (**a**) shows hyperintense content of the cystic component. Post-contrast fs T1-weighted images demonstrate progressive enhancement of the solid papillary component during the early (**b**), intermediate (**c**), and delayed (**d**) phases.

The cystic component was multiloculated and predominantly hyperintense on both T2-weighted and T1-weighted sequences, suggesting haemorrhagic or proteinaceous content (Figure 3).

A sizeable intralesional solid component with papillary morphology showed heterogeneous predominantly iso- to mildly hyperintense signal on T2-weighted imaging (Figure 2), mild diffusion restriction on DWI/ADC sequences, and progressive post-contrast enhancement (Figure 3). The lesion demonstrated expansive behaviour with marked mass effect on adjacent pelvic organs. The urinary bladder was displaced anteriorly and towards the left, the prostate gland was compressed inferiorly, and the rectal lumen appeared collapsed. No macroscopic signs of invasion of the surrounding structures were identified. The right seminal vesicle was not clearly visualised, raising suspicion for tumour origin from the seminal vesicle.

### 2.2. Urological Evaluation and Surgical Treatment

Urethrocystoscopy confirmed extrinsic compression of the bladder without evidence of intravesical lesions; therefore, the patient underwent transperineal ultrasound-guided biopsy of the pelvic mass at the referring institution. Histological analysis demonstrated a mixed epithelial and stromal proliferation without histological criteria of malignancy. The available referral documentation did not provide details regarding immunohistochemical assessment of the biopsy. The patient was subsequently referred to our institution for further evaluation and following multidisciplinary tumour board discussion, the imaging findings were considered highly suggestive of seminal vesicle cystadenoma/MEST spectrum tumour. The patient was consented and was scheduled for robotic-assisted resection of the right seminal vesicle.

The risk of radical prostatectomy was also explained. Given the potential risk of injury to the right vas deferens and following fertility preservation counselling, a testicular sperm extraction (TESE) biopsy was also performed for sperm cryopreservation.

The patient was positioned supine with a 30° Trendelenburg tilt. Robotic port placement was performed according to the standard technique, consisting of a supraumbilical 8-mm camera port for a 0° endoscope, three 8-mm robotic working ports, and a 12-mm assistant port in the right flank.

**Figure 4:**
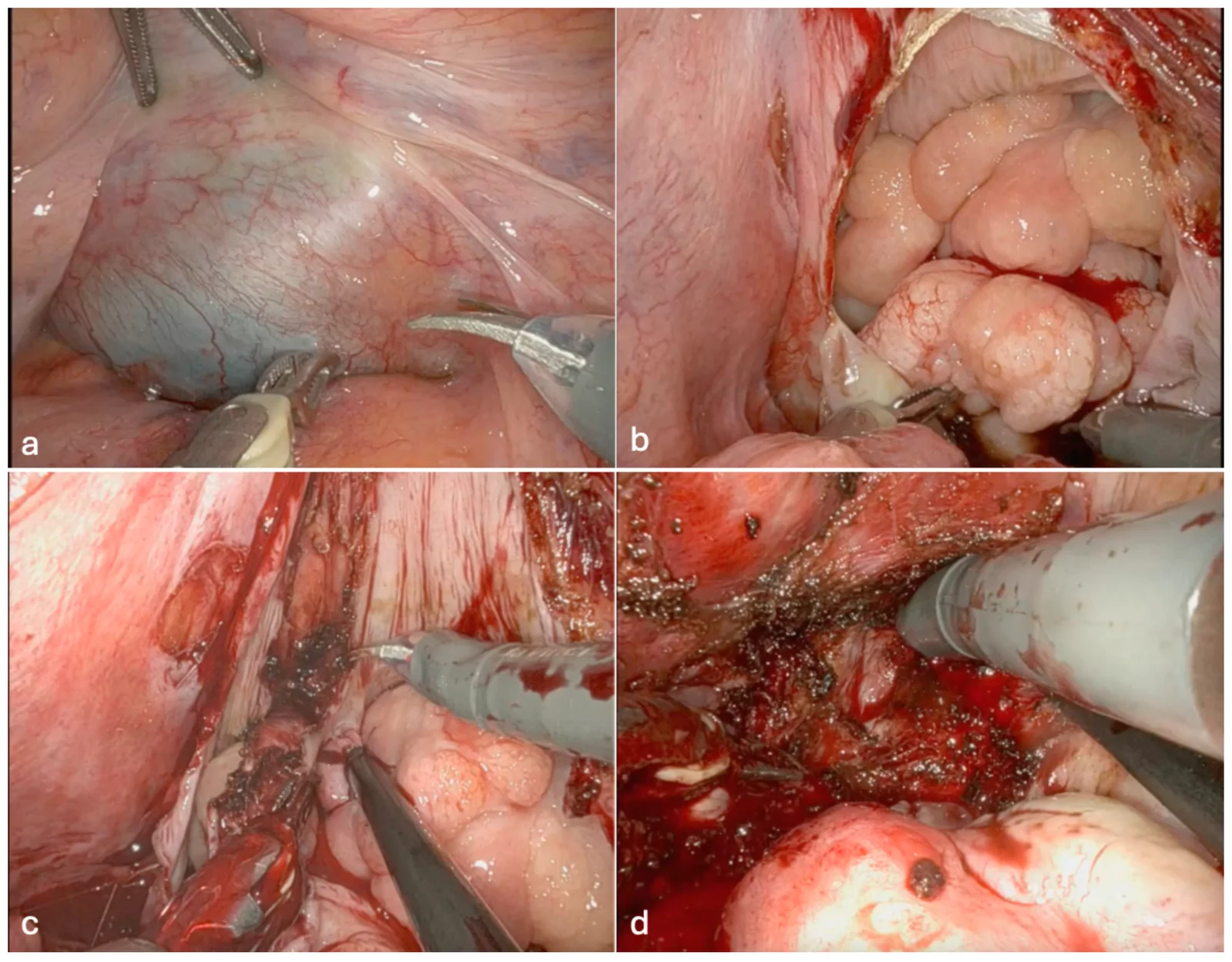
(**a**) Visualisation of the cystic mass from the abdominal cavity at the pouch of Douglas. (**b**) Fenestration of the lesion to empty the cystic fluid. (**c**) Dissection of the solid mass towards the base. (**d**) Division of the base at its attachment to the prostatic parenchyma.

Following docking of the da Vinci Xi robotic system, a posterior approach to the rectovesical space was undertaken by incising the peritoneum over the pouch of Douglas (Figure 4a). Given the large size of the cystic component, controlled fenestration was performed to facilitate exposure and mobilization of the lesion; no macroscopic spillage beyond the operative field was observed (Figure 4b).

The pedunculated mass was carefully dissected together with the right vas deferens towards its stalk, with special care taken to preserve the contralateral seminal vesicle and vas deferens (Figure 4c). Once the stalk had been completely mobilized, it was divided at its attachment to the posterior aspect of the prostate (Figure 4d), allowing en bloc removal of the lesion. The specimen was placed in an endoscopic retrieval bag. Hemostasis was confirmed, and a topical hemostatic agent was applied to the resection bed. No evidence of injury to the rectum, bladder, or contralateral seminal vesicle was identified.

The patient had an uneventful postoperative recovery. The Foley catheter was removed after a successful trial of void, and the patient was discharged on postoperative day 2.

### 2.3. Pathological and Immunohistochemical Findings

**Figure 5:**
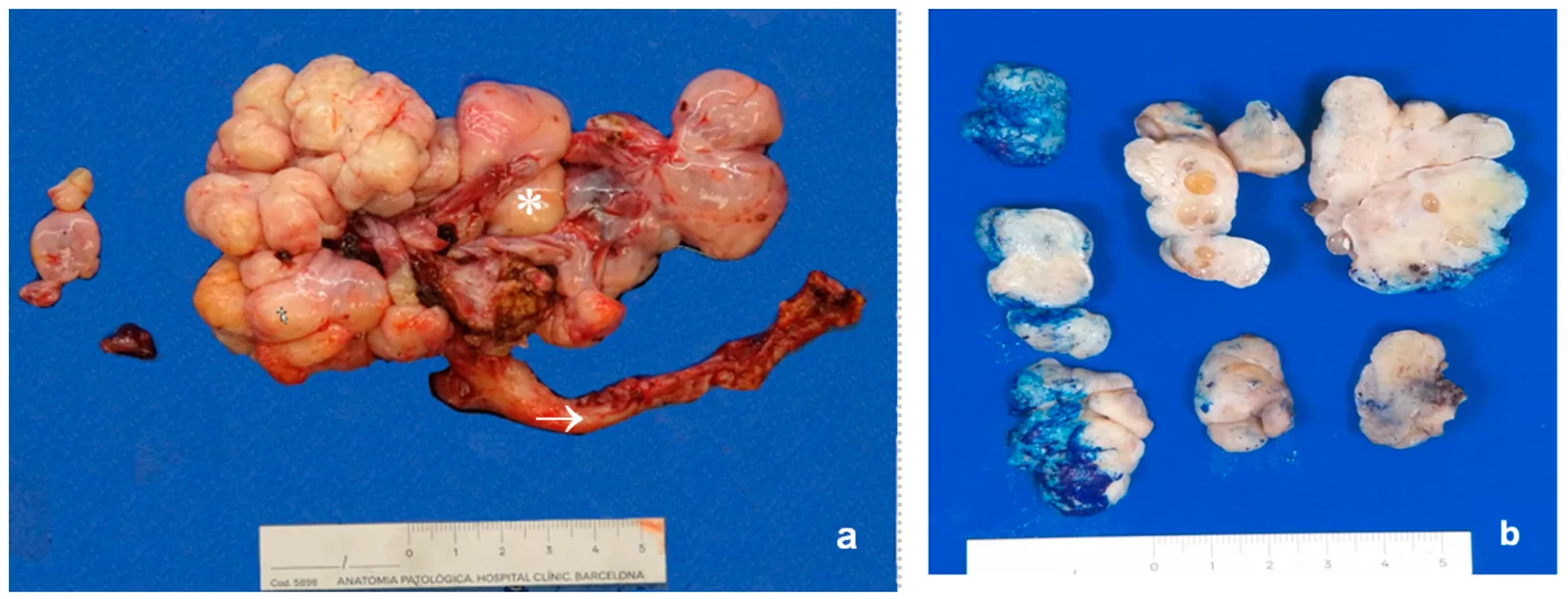
(**a**) Gross pathological examination revealing a white multiloculated lesion (asterisk), with a smooth external surface; and the vas deferens (arrow). (**b**) Gross pathological sections, with solid and cystic areas of the multiloculated lesion.

Gross pathological examination revealed a seminal vesicle and vas deferens containing a multiloculated lesion with solid and cystic areas. The cystic areas were filled with clear viscous material, and the solid areas showed a white fibrotic homogeneous surface (Figure 5).

**Figure 6:**
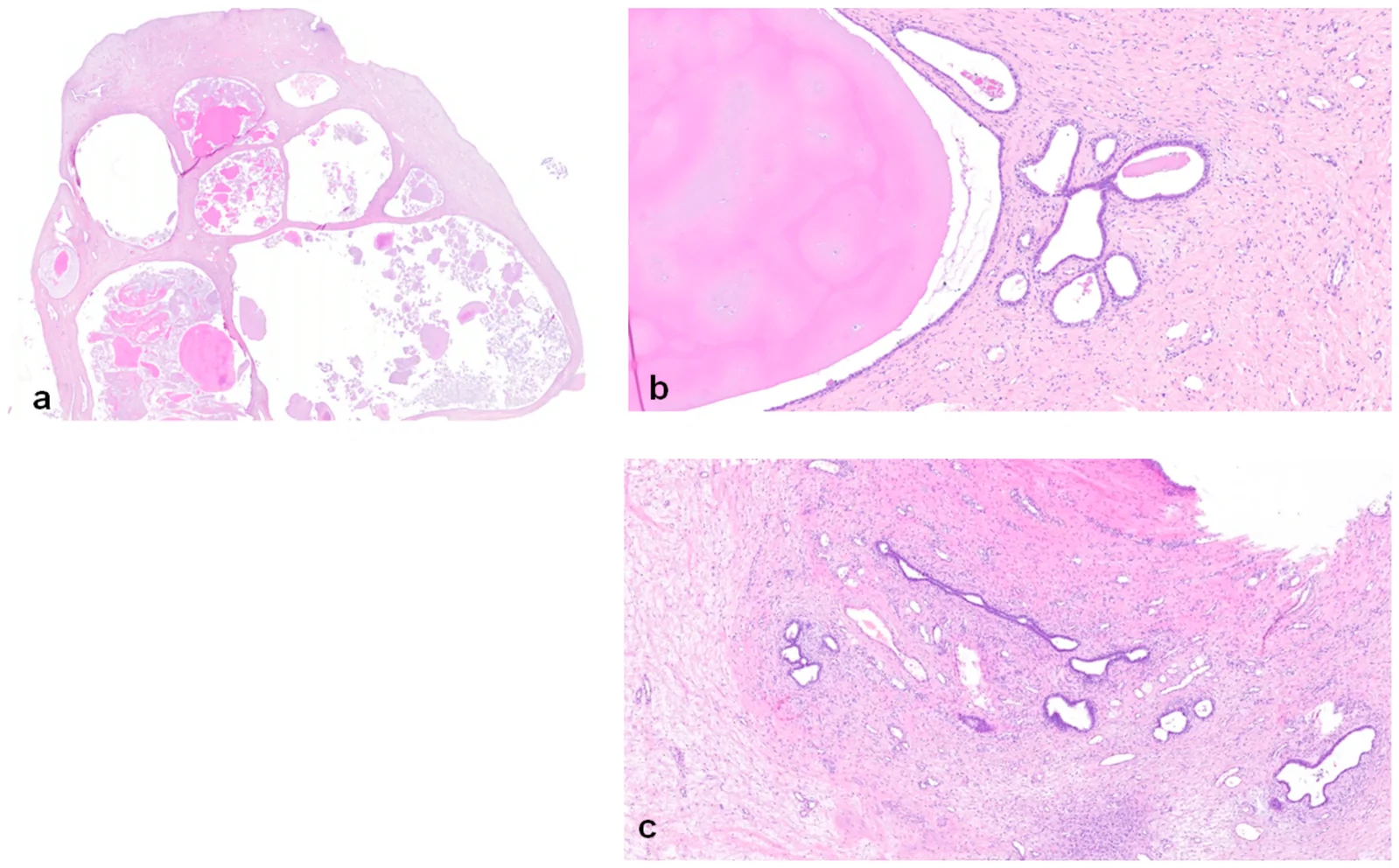
Hematoxylin and eosin-stained sections. (**a**) Whole slide image (×0.5). (**b**) The tumour shows a biphasic histology, with unilocular cysts lined by cuboidal cells and surrounded by the stromal component (×5). (**c**) The stromal component is composed of spindle cells, focally hypercellular, with no evidence of necrosis, atypical nuclei or mitotic activity (×5).

**Figure 7:**
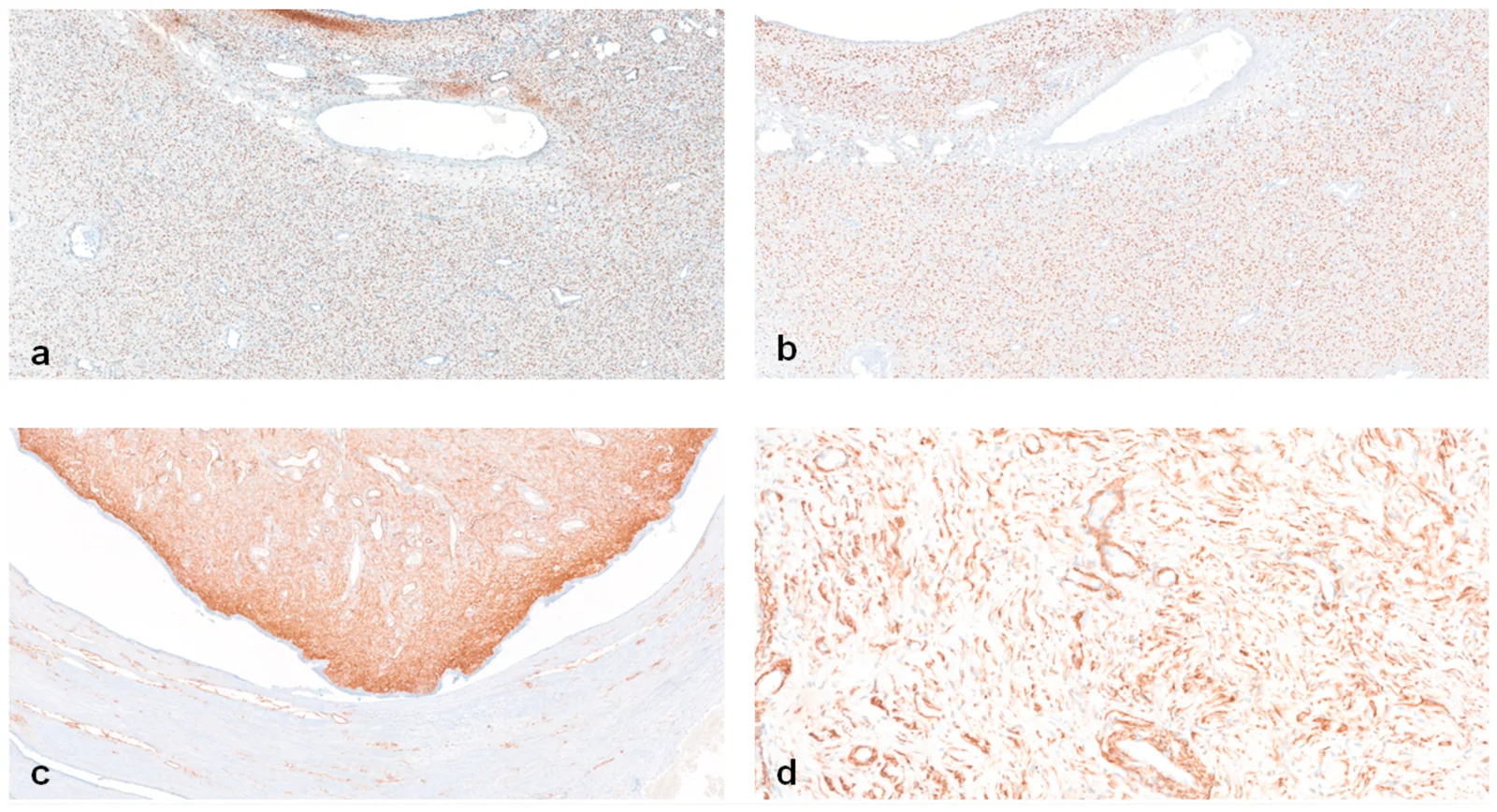
Immunohistochemical staining of the stromal component. (**a**,**b**) ER and PR show diffuse nuclear staining in the stromal component (×5). (**c**,**d**) SMA and CD34 show diffuse cytoplasmic and membranous staining, respectively, in the stromal component (×1 and ×10).

Histological analysis revealed a biphasic lesion with stromal and epithelial cellularity. The epithelial component was constituted of cysts with an epithelial lining of cuboidal cells, with a low nuclear/cytoplasmic ratio lacking atypia. The cysts were unilocular, without intraluminal epithelial projections and were surrounded by focally hypercellular spindle cells, with no evidence of necrosis, atypical nuclei, or mitotic activity, constituting the stromal component (Figure 6). The surgical margins were free of tumour. Immunohistochemistry showed positivity for oestrogen receptor (ER), progesterone receptor (PR), smooth muscle actin (SMA) and CD34 in stromal cells (Figure 7). In some sections, prostatic parenchyma was identified, confirmed by NKX3.1 and PSA immunostaining. GATA3 and CK7 were positive in the entire epithelial component which is compatible with a Wolffian/seminal vesicle origin in the appropriate morphological and anatomical context. NKX3.1, PSA, c-KIT and calretinin were negative in the neoplastic component, excluding a prostatic or mesothelial origin. At the surface of the lesion, the stromal component exhibited a lobulated or polypoid configuration, with an external lining of flattened epithelial-like cells [10].

These findings confirmed the diagnosis of a seminal vesicle mixed epithelial-stromal tumour (MEST). The absence of stromal atypia, necrosis, and mitotic activity is consistent with the low-grade morphology described in previously proposed grading schemes. However, these grading schemes have not been widely validated or formally adopted, and their prognostic significance remains uncertain given the limited number of reported cases [4,8].

### 2.4. Outcome and Follow-Up

The postoperative clinical course was uneventful, and the patient remained asymptomatic at follow-up. MRI performed 6 months after surgery showed only residual fibrotic changes at the surgical site, with no evidence of residual or recurrent tumour. The relatively short follow-up period of six months represents a limitation of the present report and does not allow conclusions regarding long-term recurrence risk. Long-term clinical and radiological surveillance is therefore planned.

## 3. Differential Diagnosis

The differential diagnosis of a large retrovesical cystic pelvic mass in a young male is broad and includes seminal vesicle cystadenoma, Müllerian duct cysts, ejaculatory duct cysts, prostatic cystic neoplasms, seminal vesicle adenocarcinoma, retrovesical sarcoma and prostatic stromal tumours. In the present case, the relatively prominent enhancing solid component and complex internal architecture were considered atypical for a conventional seminal vesicle cystadenoma [11,12] and should orient towards the diagnosis of an epithelial-stromal neoplasm within the MEST/cystadenoma spectrum. Moreover, the predominantly expansile growth pattern favoured a benign or low-grade lesion, helping to distinguish it from more aggressive entities such as seminal vesicle adenocarcinoma and retrovesical sarcomas. Primary seminal vesicle adenocarcinomas are more frequently associated with hematospermia or hematuria and demonstrate an infiltrative behaviour with a more complex solid component, which may contain necrotic areas and exhibit ill-defined margins [13,14].

**Figure 8:**
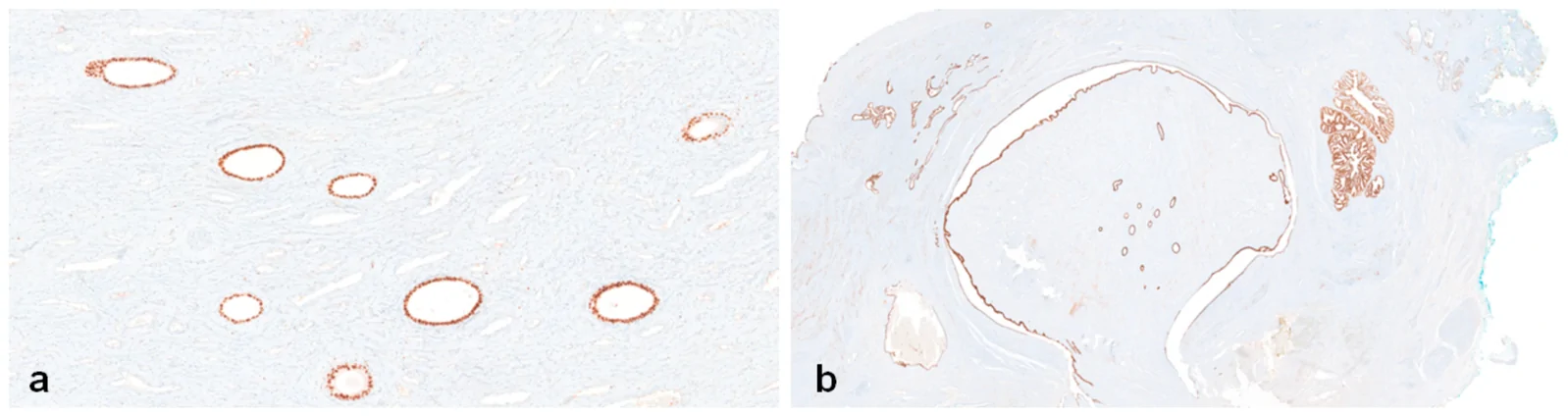
Immunohistochemical staining of the epithelial component. (**a**) GATA3 shows diffuse nuclear staining in the epithelial component (×5). (**b**) CK7 shows diffuse cytoplasmic staining in the epithelial component (×1).

Histologically, seminal vesicle MEST has morphological overlap with prostate STUMP. Prostatic STUMP (stromal tumour of uncertain malignant potential) should be excluded using immunohistochemistry studies for NKX3 and PSA (both being positive in prostate glands) and CK7 and PAX8 (negative in prostate origin). Stromal component may be very similar in both lesions with positivity for CD34, vimentin, desmin and actin, but seminal vesicle MEST shows positivity to hormonal receptors (ER and PR), whereas prostate STUMP is positive only for PR. The combined immunophenotype of the epithelial component with positivity for GATA3 and CK7 supports the seminal vesicle origin (Figure 8). Once the seminal vesicle origin is determined, the main differential diagnosis is between cystadenoma and MEST. Cystadenoma typically presents as a predominantly cystic lesion with a monolayer or bistratified epithelial cells without atypia, and a cystic wall composed mainly of muscle cells [4].

## 4. Discussion

MEST is a rare biphasic neoplasm composed of epithelial and stromal components. While the kidney is the most common site of origin, involvement of the seminal vesicle is extremely uncommon. Since the first reported cases, terminology has remained heterogeneous, with lesions variably classified as cystadenoma, epithelial-stromal tumour or phyllodes-like tumour.

Clinical presentation is often related to mass effect on adjacent pelvic organs. Reported symptoms include urinary frequency, dysuria, haematuria, pelvic pain, infertility and urinary retention. In the present case, the clinical presentation was somewhat atypical, as the patient experienced recurrent episodes of acute urinary retention without other typical symptoms previously described in association with seminal vesicle MEST, such as hematuria or pelvic pain. Although this symptom is nonspecific, recognition of the possibility of a seminal vesicle mass in patients with recurrent obstructive symptoms may facilitate appropriate pelvic imaging and subsequent diagnosis.

Radiologically, seminal vesicle MESTs typically appear as large retrovesical multiloculated cystic masses containing variable solid stromal components. MRI is particularly valuable for lesion characterisation and evaluation of local extension. The cystic component may demonstrate T1 hyperintensity due to haemorrhagic or proteinaceous content, while the solid component often exhibits heterogeneous enhancement reflecting stromal cellularity. The absence of frank local invasion and the expansive rather than infiltrative growth pattern may help distinguish these lesions from aggressive malignancies. Although preoperative diagnosis remains difficult because imaging findings overlap with those of seminal vesicle cystadenoma and other retrovesical cystic lesions, several imaging features suggested a more complex epithelial-stromal neoplasm. Classical seminal vesicle cystadenomas are predominantly cystic, well-circumscribed lesions with thin septations and minimal or absence of solid enhancing components. In our case, the presence of a relatively prominent solid component with progressive enhancement, subtle diffusion restriction and haemorrhagic/proteinaceous cystic content may raise suspicion for a lesion within the MEST spectrum rather than a simple cystadenoma [6,7,8,9]. Importantly, the value of the present case lies in the integrated correlation between the multiparametric MRI findings and the surgical and histopathological features. The multiloculated cystic architecture corresponded to the epithelial-lined cystic component, while the enhancing papillary solid component correlated with the stromal and epithelial proliferation identified histologically. The absence of macroscopic invasion on MRI was also concordant with the low-grade histological features and the expansile growth pattern observed at surgery. This radiologic-pathologic correlation provides a more comprehensive characterisation of the imaging phenotype of seminal vesicle MEST and may help radiologists recognize this rare epithelial-stromal neoplasm when evaluating a complex retrovesical mass.

The 2004 WHO Classification distinguished between cystadenomas and MESTs based on the presence of neoplastic stroma in MEST; however, several authors have suggested that many lesions historically classified as seminal vesicle cystadenomas may belong to the MEST spectrum, reflecting evolving pathological classification systems. Although some authors proposed unified grading of MEST tumours (low, intermediate and high grade) regarding stromal atypia, mitotic activity, pleomorphism and necrosis, these have not been widely adopted [4,5].

Most of the reported cases would fall in the category of low or intermediate grade, while high grade ones are even more exceptionally rare, being the ones associated with aggressive characteristics and metastatic potential. The pathogenesis of these tumours remains not completely understood, although the expression of ER and PR may suggest that hormonal activity plays a role, bearing in mind the embryological derivation of the seminal vesicles, which share similarities with Müllerian structures. Notably, this entity remains exceedingly rare, with only approximately a dozen cases reported in the WHO Classification [10]. The biological behaviour of these tumours remains incompletely characterized [9]. Because of the rarity of the disease, no standardised management guidelines exist. Complete surgical excision remains the treatment of choice and is generally associated with favourable prognosis in lesions without malignant transformation. The robotic approach is particularly advantageous in the deep pelvic field, allowing better visualisation, precise dissection and meticulous preservation of the surrounding anatomical structures. Previous reports have described different surgical approaches, including local tumour excision, seminal vesiculectomy, cystectomy, and radical prostatectomy. The choice of procedure was determined by the tumour’s histological grade, size, and extent of local invasion. The prognosis is generally excellent for benign and low-grade tumours. There have been few reported series of malignant high grade MEST tumours with metachronous recurrence or lung metastasis [15,16]. Given the potential for delayed local recurrence, long-term urological follow-up is recommended.

Although MEST of the seminal vesicle is exceedingly rare, the relevance of the present case extends beyond reporting an additional occurrence of this uncommon tumour. To our knowledge, this case provides a detailed multiparametric MRI description with direct radiologic-pathologic correlation, allowing the imaging appearance to be interpreted in relation to the underlying epithelial and stromal components. This correlation may contribute to a better understanding of the imaging phenotype of seminal vesicle MEST and may help include this entity in the differential diagnosis of unusual seminal vesicle and pelvic masses.

## 5. Conclusions

MEST of the seminal vesicle is an exceptionally rare biphasic neoplasm that should be considered in the differential diagnosis of a large complex retrovesical mass in young male patients, particularly when a prominent enhancing solid component is present. In the present case, multiparametric MRI provided detailed characterisation of the multiloculated solid-cystic architecture, haemorrhagic/proteinaceous content, progressive enhancement of the solid component, and expansive relationship with the adjacent pelvic organs. Importantly, the imaging findings showed direct correlation with the epithelial and stromal components identified at histopathological examination, providing an integrated radiologic-pathologic characterization of this rare entity.

Although these imaging features are not pathognomonic, recognition of this pattern may help raise suspicion for a seminal vesicle MEST or related epithelial-stromal neoplasm and guide the differential diagnosis and surgical planning. Definitive diagnosis relies on histopathological and immunohistochemical evaluation. Complete surgical excision remains the treatment of choice, with generally favourable outcomes for low-grade lesions, although the rarity of this tumour and reports of recurrence underline the importance of long-term follow-up.

## Data Availability

The data presented in this study are available from the corresponding authors upon request.

## Abbreviations

The following abbreviations are used in this manuscript:

ADC	Apparent diffusion coefficient
CD34	Cluster of differentiation 34
CK7	Cytokeratin 7
CT	Computed tomography
DWI	Diffusion-weighted imaging
ER	Oestrogen receptor
GATA3	GATA binding protein 3
MEST	Mixed epithelial stromal tumour
MRI	Magnetic resonance imaging
NKX3.1	NK3 homeobox 1
PAX8	Paired box 8
PR	Progesterone receptor
PSA	Prostate-specific antigen
SMA	Smooth muscle actin
STUMP	Stromal tumour of uncertain malignant potential
TESE	Testicular sperm extraction
WHO	World Health Organization
c-KIT	KIT receptor tyrosine kinase
